# Mesoscale characterization of osseointegration around an additively manufactured genistein-coated implant

**DOI:** 10.1038/s41598-024-66058-1

**Published:** 2024-07-03

**Authors:** Chiara Micheletti, Liza-Anastasia DiCecco, Joseph Deering, Wanqi Chen, Ana Cláudia Ervolino da Silva, Furqan A. Shah, Anders Palmquist, Roberta Okamoto, Kathryn Grandfield

**Affiliations:** 1 Department of Materials Science and Engineering, McMaster University, Hamilton, ON Canada; 2 Department of Biomaterials, Sahlgrenska Academy, University of Gothenburg, Gothenburg, Sweden; 3 Department of Diagnosis and Surgery, Araçatuba Dental School, São Paulo State University, Araçatuba, SP Brazil; 4 Department of Basic Sciences, Araçatuba Dental School, São Paulo State University, Araçatuba, SP Brazil; 5 School of Biomedical Engineering, McMaster University, Hamilton, ON Canada; 6 Brockhouse Institute for Materials Research, McMaster University, Hamilton, ON Canada; 7 Research Productivity Scholarship (Process: 309408/2020-2), Araçatuba, SP Brazil

**Keywords:** Mineral ellipsoid, PFIB-SEM tomography, Scanning transmission electron microscopy, Resin cast etching, LCN, Osseointegration, Implants, Microscopy

## Abstract

Given the hierarchical nature of bone and bone interfaces, osseointegration, namely the formation of a direct bone-implant contact, is best evaluated using a multiscale approach. However, a trade-off exists between field of view and spatial resolution, making it challenging to image large volumes with high resolution. In this study, we combine established electron microscopy techniques to probe bone-implant interfaces at the microscale and nanoscale with plasma focused ion beam-scanning electron microscopy (PFIB-SEM) tomography to evaluate osseointegration at the mesoscale. This characterization workflow is demonstrated for bone response to an additively manufactured Ti-6Al-4V implant which combines engineered porosity to facilitate bone ingrowth and surface functionalization via genistein, a phytoestrogen, to counteract bone loss in osteoporosis. SEM demonstrated new bone formation at the implant site, including in the internal implant pores. At the nanoscale, scanning transmission electron microscopy and energy-dispersive X-ray spectroscopy confirmed the gradual nature of the bone-implant interface. By leveraging mesoscale analysis with PFIB-SEM tomography that captures large volumes of bone-implant interface with nearly nanoscale resolution, the presence of mineral ellipsoids varying in size and orientation was revealed. In addition, a well-developed lacuno-canalicular network and mineralization fronts directed both towards the implant and away from it were highlighted.

## Introduction

Bone bonding to titanium implants is a multiscale phenomenon, spanning from a macro-level of anchorage to the microscale where cells interact with the implant surface, all the way down to the nanoscale where the titanium oxide surface layer intermixes with calcium and phosphorus^1^. Originally, bone-implant contact, i.e., “osseointegration”, was defined “on the light microscopic level”^2^. The advent of scanning/transmission electron microscopy (S/TEM) imaging of bone-implant interfaces, combined with minimally destructive sample preparation by focused ion beam (FIB)^3^, highlighted that osseointegration is also a nanoscale phenomenon^4–6^. While various ultrastructural models for the bone-implant interface have been proposed (reviewed in^1^), STEM and electron tomography have demonstrated direct bone bonding to nanotextured titanium-based implants in both two dimensions (2D) and three dimensions (3D), respectively^4,7,8^. Over the years, it has thus emerged that a multiscale and multimodal characterization approach is ideal to evaluate osseointegration and bone-biomaterial interfaces in a comprehensive manner^6,9^, where the term “interface” refers to “the bone-implant boundary and a limited volume of peri-implant bone within which strain fields arising from implant loading radiate outward for a considerable distance, believed to be about one implant radius”^1^.

That bone-implant interfaces are best studied across multiple length scales is not surprising, given the hierarchical nature of bone itself. This multi-level architecture begins with the distinction between cortical and trabecular within the whole bone at the macroscale, and culminates with the mineralized collagen fibrils, and other small proteins and water, at the nanoscale, passing through other levels of organization such as osteons in cortical bone^10^. While in the words of Richard Feynman *“there’s plenty of room at the bottom”*, a renewed interest in studying bone at the mesoscale, i.e., in between micro- and nano-levels, has recently emerged^11,12^, especially thanks to the implementation of FIB-scanning electron microscopy (SEM) tomography in bone research^13^. FIB-SEM tomography probes relatively large 3D volumes of material, typically in the order of tens of microns for Ga^+^ FIB instruments, with a spatial resolution on the nanoscale level. The volume of material examined can be further expanded to hundreds of microns by the use of Xe^+^ plasma FIB (PFIB) instruments, without compromising the resolution^14^. This opens new avenues to evaluate bone-implant interfaces over greater distances from the implant surface, as recently demonstrated for an additively manufactured implant with engineered porosity^15^. Not only can PFIB-SEM tomography characterize osseointegration over large volumes, but it is also well suited to probe the mesoscale structure of newly formed bone, particularly to examine mineral ellipsoids (or “tesselles”), i.e., ellipsoidal-shaped mineral assemblies 0.5–1 µm in diameter and 2–2.5 µm in length^11,12^. Mineral ellipsoids have been only recently included in the mesoscale architecture of bone, and had previously gone undetected in many studies. However, in hindsight, they have been identified as “rosettes”^16^ and “elliptical motifs”, i.e., their 2D transverse and longitudinal cross-sections, respectively, at bone-biomaterial interfaces^17^. As their presence appears ubiquitous across species, anatomical locations, and implant materials^17^, it appears important to implement the study of bone’s mesoscale architecture in the multimodal and multiscale characterization platforms used in osseointegration research.

Herein, multiscale evaluation of osseointegration encompassing the mesoscale is applied to bone interfacing with a porous Ti-6Al-4V implant manufactured by laser powder bed fusion (L-PBF) and surface-modified by acid-etching followed by functionalization with a layer-by-layer genistein coating. This implant was selected as a model to demonstrate the applicability of our characterization platform to additively manufactured implants subjected to physico-chemical surface modifications and in the presence of systemic conditions altering bone repair (ovariectomy). However, a quantitative evaluation of its biological performance, especially regarding the role of the genistein coating, was beyond the scope of this work. Overall, this implant design combines the advantages of engineered porosity in implants with those of local drug delivery to improve osseointegration in osteoporotic conditions. While osteoporosis is not considered a contraindication to the installation of bone implants, systemic conditions affecting bone quantity and/or quality can impair osseointegration^18^. Genistein is a natural phytoestrogen capable of promoting osteoblastogenesis^19^ and increasing bone mass^20^, while avoiding the side effects of antiresorptive medications, such as bisphosphonate-related osteonecrosis of the jaw^21^. Additionally, porous implants can promote cell migration and angiogenesis, and stimulate bone ingrowth, which has been shown to increase bone-implant stabilization^22–24^. Porous implants can be fabricated by additive manufacturing to afford great flexibility in terms of implant design, including pore volume, size, and distribution^25,26^.

The multiscale and multimodal characterization approach we present aims to examine not only the bone-implant interface, but also the structure of newly formed bone and its cellular network. Our characterization platform encompasses SEM, to gain an overview of bone formation in the peri-implant space and cell-implant interactions, high-angle annular dark-field (HAADF)-STEM, to resolve the nanoscale bone-implant bonding, and PFIB-SEM tomography to bridge micro- and nanoscale to examine both the mesoscale architecture of newly formed bone and the lacuno-canalicular network (LCN) within.

## Results and discussion

### Microscale: bone-implant contact and LCN

**Figure 1:**
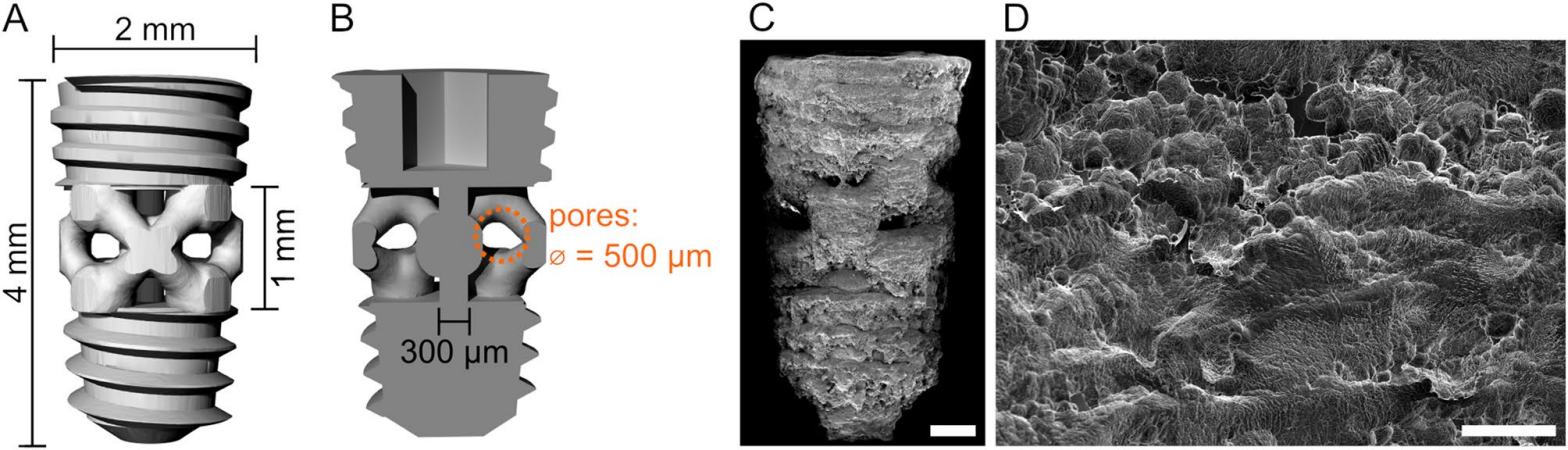
Implant design and final surface topography. (**A**) 3D rendering of the implant design. (**B**) Longitudinal cross-section of the implant. The main dimensions of the implant and pores are reported in A and B. (**C**) Overview SEM image (secondary electron mode) of the implant after acid etching. (**D**) Higher magnification SEM image of the implant surface topography after acid etching. Scale bars are 500 µm in C and 50 µm in D.

Ti-6Al-4V implants with engineered porosity in their mid-section were manufactured by L-PBF (Fig. 1), followed by acid etching and surface functionalization with genistein by an electrochemical layer-by-layer coating technique [patent application BR 10 2021 019,134 1, Instituto Nacional da Propriedade Industrial, Brazil]. Additively manufactured bone implants with pore sizes spanning from 50 µm to 1500 µm and porosity levels from 30 to 70% have been reported in the literature^24^. This inconsistency in implant design can be attributed to variability in animal models, anatomical sites, and physico-chemical characteristics of the implant surface. Herein, we selected a pore size of 500 µm as the range 300–500 µm typically shows good osseointegration^24^. While the optimal level of porosity remains difficult to establish, especially given the dissimilar experimental conditions, the porosity we selected (45%) guaranteed adequate mechanical integrity of the implant during its placement in the rat tibia.

Additive manufacturing techniques based on powder bed fusion such as L-PBF produce implants with a micro-rough surface finish due to the presence of partially unmelted/unsintered raw powders^27,28^. In this study, the as-printed implant surface roughness was in the order of a few tens of micrometres, given the size of the feedstock powders used (15–45 µm). The post-printing acid etching treatment preserved some of the unmelted/unsintered particles, at the same time partially smoothening the surface, and thus creating additional features on the submicron scale, as shown in Fig. 1C,D.

**Figure 2:**
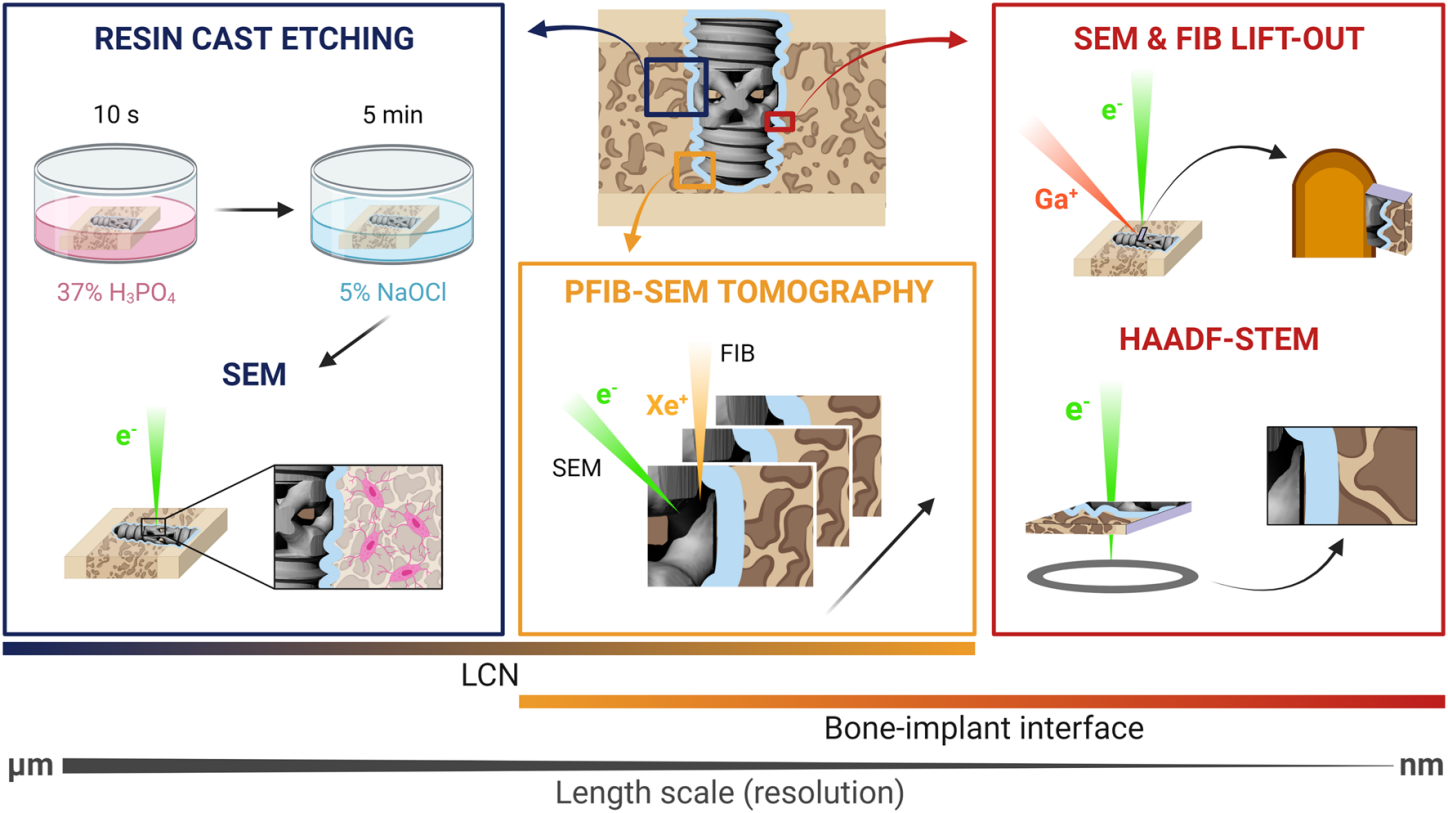
Schematic (not to scale) of the multimodal and multiscale characterization workflow of the bone-implant interface and the LCN. The blue border around the porous implant represents the genistein coating. The LCN was examined in 2D by SEM imaging after resin cast etching and in 3D by PFIB-SEM tomography. PFIB-SEM tomography was also used to image the bone-implant interface. Nanoscale resolution was achieved by HAADF-STEM imaging of an electron transparent lamella prepared by the FIB in situ lift-out technique.

After the in vivo study in ovariectomized rats, the bone-implant interface and the LCN were characterized using the multimodal, i.e., combining different techniques, and multiscale, i.e., going from the micro- to the nanoscale, approach schematically illustrated in Fig. 2.

**Figure 3:**
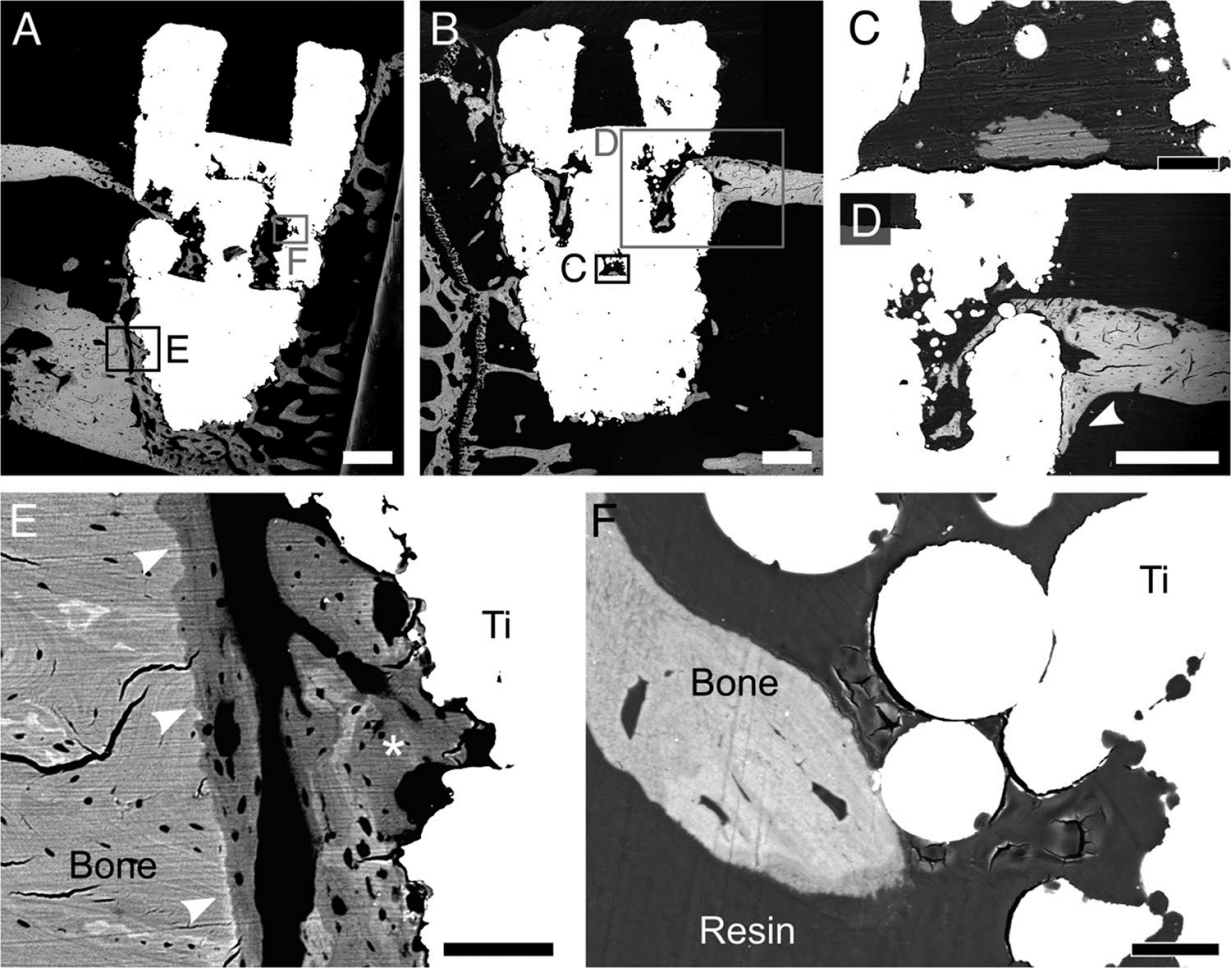
BSE-SEM images of the peri-implant space and the bone-implant interface. (**A**-**B**) BSE-SEM mosaic images of two genistein-coated Ti-6Al-4V porous implants and their peri-implant space 28 days after implant placement. (**C**) Magnified image of bone within the porous space. (**D**) New bone growing from the native upper cortex down towards the implant (marked by arrowhead). (**E**) New bone growing from pre-existing cortical bone in the lower cortex (marked by arrowheads) and from the implant surface (label “Ti”) (marked by the asterisk), indicating distance and contact osteogenesis, respectively. A difference in greyscale level can be noted between old (lighter) and new (darker) bone. (**F**) Bone formed within the porous space in close association with microparticles from L-PBF. Scale bars are 500 µm in A, B, and D, 50 µm in C, 100 µm in E, and 20 µm in F.

Characterization of osseointegration commonly begins at the microscale to gain information on new bone formation around an implant. Herein, this was accomplished by backscattered electron (BSE)-SEM imaging to easily distinguish new *vs.* old bone thanks to *Z* (atomic number)-contrast. New bone was detected both in proximity to the exterior implant surface (Fig. 3A,B), as well as inside the porous space (Fig. 3C), indicating bone ingrowth facilitated by the porous design. As new bone is continually produced over time, the continuous ingrowth inside the porous space would provide a greater surface area for bone-implant contact and increased interlocking, creating a strong bone-implant interface^23^.

Areas of new bone originating at the native cortical bone were noted as lower *Z*-contrast regions connected to higher *Z*-contrast regions, representing new and relatively older tissue, respectively (Fig. 3D,E). These areas, together with new bone close to the implant surface and located away from the existing tissue, indicate that bone formation proceeds via both distance and contact osteogenesis^29^. Contact osteogenesis is especially apparent within the implant porous space (Fig. 3F). Although not possible to chemically confirm its presence in vivo in this study, this new bone formation, especially considering the compromised bone repair in the ovariectomized rodent^30^, may be in part attributed to the genistein coating, since genistein has a positive effect on osteoblasts^19^, hence on bone formation.

New bone formed in close contact with leftover microparticles from L-PBF was observed (Fig. 3F), demonstrating interaction with the surface microtopography, as well as the additional submicron-texturing created by acid etching (Fig. 1C,D). This is consistent with several studies showing that surface topography presenting micro-to-nano features favours osseointegration as it provides mechanical interlocking between bone and implant, creates a greater surface area for protein adsorption, and favours cell interactions^31,32^.

**Figure 4:**
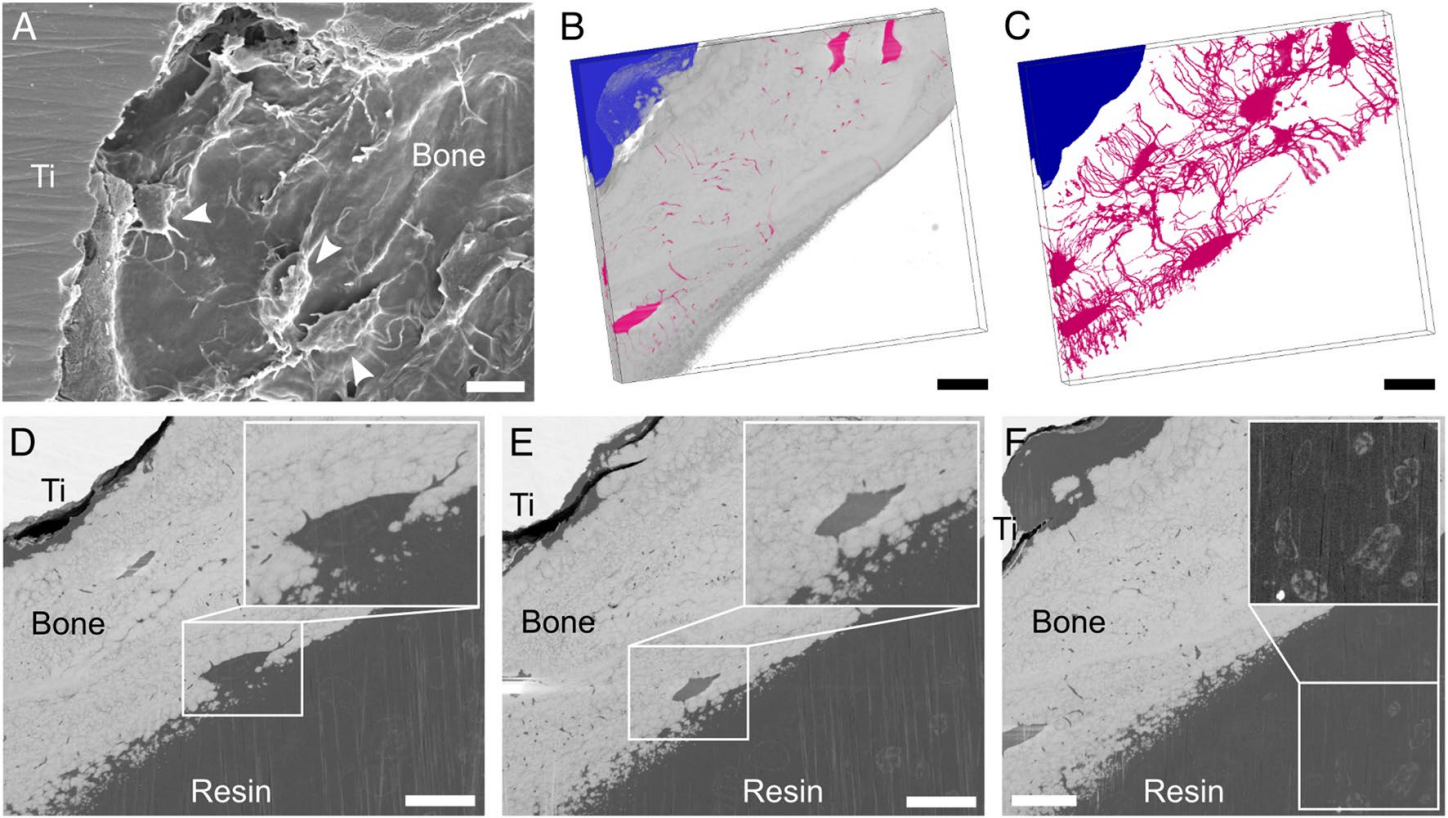
LCN at the bone-implant interface. (**A**) SEM image (secondary electron mode) of a sample after resin cast etching, where cells (marked by arrowheads) and their processes are visible in bone juxtaposed to the Ti-6Al-4V implant (indicated by the label “Ti”). (**B**-**C**) Visualization of the PFIB-SEM tomogram with (B) and without (C) bone (light grey), where implant and LCN are coloured in blue and pink, respectively (resin not shown in the rendering, but available in Fig. S1-B). (**D**-**E**) Sequence of slices in the PFIB-SEM dataset showing a cell becoming entrapped within the mineralizing bone matrix (magnified in insets). While in D part of the lacuna cannot be clearly distinguished from the resin, the same lacuna is fully surrounded by bone matrix in E. The image planes in D and E are approximately 5.8 µm away from each other in the image stack. (**F**) Some brighter features, possibly corresponding to membrane-bound intracellular organelles, can be noted in the resin, especially in the bottom right corner (contrast-enhanced in the inset). Scale bars are 10 µm.

As osteoblasts become entrapped within the mineralizing matrix, differentiating into osteocytes^33^, an extensive LCN develops in newly formed bone. The LCN plays an essential role in mechanosensing^34,35^, and cellular morphology and interconnectivity can reveal important information on bone quality and bone response to specific biomaterials^36^. The formation of an interconnected network of osteocytes was visualized by resin cast etching (Fig. 4A), which is a facile method to expose the LCN and directly visualize it by SEM^37–39^. However, despite the 3D-like appearance of SEM images post-resin cast etching, such images are inherently 2D. Therefore, the presence of a well-developed LCN was further confirmed in 3D by PFIB-SEM tomography (Figs. 4B,C, S1). Compared to conventional FIB-SEM with Ga^+^ ion sources, larger volumes of material can be probed by Xe^+^ PFIB-SEM^11,14^. A 72.95 × 63.56 × 18.93 µm^3^ volume was examined, allowing the visualization of the bone-implant interface up to 40–50 µm away from the implant surface. A total of 7 osteocyte lacunae with their canaliculi were clearly distinguishable in the dataset. Most lacunae were aligned with their major axes parallel to each other and to the implant surface, with most canaliculi running in the orthogonal direction across the bone volume. Cell processes appeared to extend towards the implant surface (Fig. 4C), similar to what has been reported by others in osseointegrated implants^38,40^.

Interestingly, one lacuna only partly enclosed by bone was noted close to the bone-resin interface (Fig. 4D,E, Video S1). This likely corresponds to a cell transitioning from osteoblast to osteocyte during bone matrix mineralization^33^. Within said lacuna, some brighter features, consisting of a circular/oval line around some globular elements, could be noted. Similar high-contrast features were also present within the resin space (Fig. 4F), analogously to what has been reported in previous work^15^. While these features resemble membrane-bound structures such as intracellular organelles, since the sample is not stained it is unclear why they display greyscale levels higher than resin and closer to mineralized bone.

### Mesoscale: mineralization front and mineral ellipsoids

**Figure 5:**
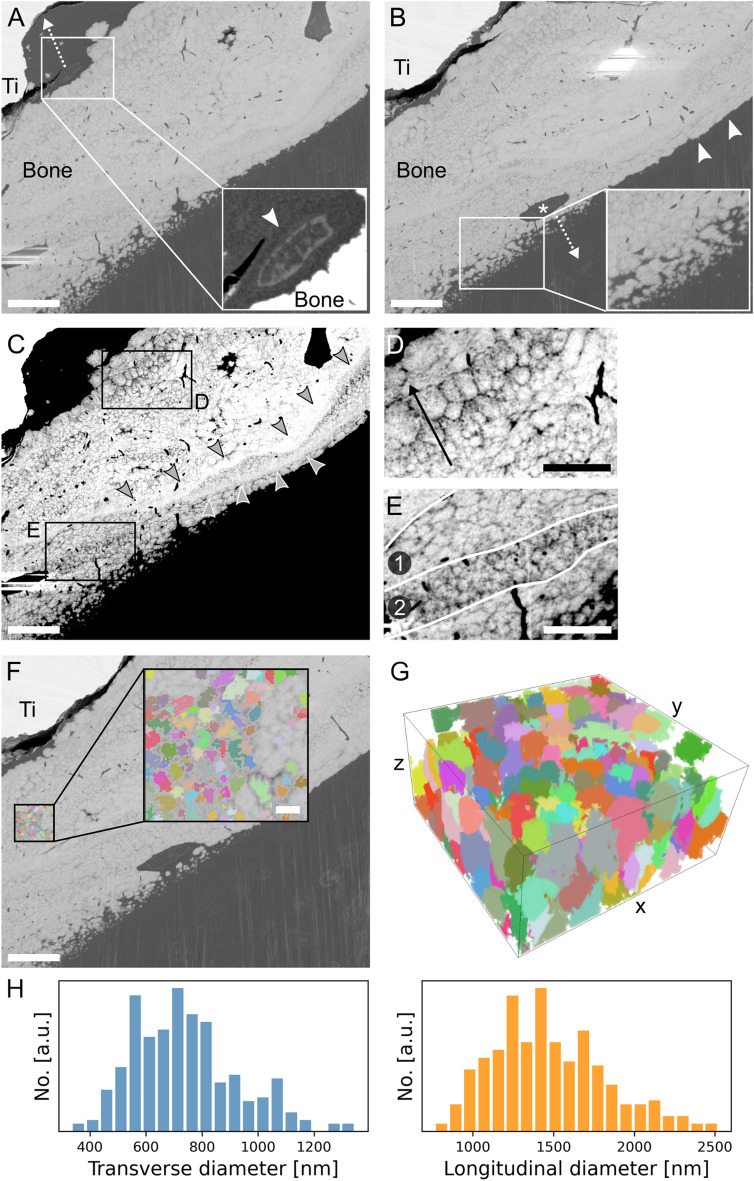
Mineral ellipsoids in newly formed bone. (**A**) A high-contrasting feature in the resin region between bone and implant (“Ti” label) marked by the arrowhead in the inset (brightness/contrast-enhanced). If this feature corresponds to a cell (presumably an osteoblastic osteocyte), this suggests that bone is growing towards the implant, as indicated by the dashed arrow. (**B**) A cell (presumably an osteoblastic osteocyte, marked by *) becoming entrapped by mineralizing bone matrix and the presence of mineral foci (enlarged in the inset) suggest that in this area bone is growing away from the implant, in the direction of the dashed arrow. Away from the cell but along the same bone surface, mineral foci are absent, and the surface appears smoother (arrowheads). (**C**) Contrast/brightness-enhanced version of image A to better visualize the mineral ellipsoids. (**D**) Magnified image showing the size variation of the ellipsoids (becoming larger in the direction of the arrow). (**E**) Magnified image exemplifying an abrupt variation in orientation of the ellipsoids from region 1, where they are predominantly cross-sectioned longitudinally (elliptical motif), to region 2, where they are predominantly cross-sectioned transversally (rosette). Arrowheads indicate two highly-mineralized bands, where no ellipsoids can be distinguished. (**F**) Region where mineral ellipsoids were segmented by the Watershed algorithm, magnified in the inset where they appear as “rosettes” (transverse cross-sections). (**G**) 3D rendering of the segmented mineral ellipsoids, where their rosette and ellipsoidal shapes are visible in the transverse (xy) and longitudinal (yz, xz) planes, respectively. (**H**) Distribution of transverse (left, blue) and longitudinal (right, orange) diameters of the mineral ellipsoids (y axis corresponds to the number of ellipsoids). Scale bars are 10 µm in A, B, C, and F, 5 µm in D and E, and 5 µm in the inset in F. The box in G is 7.5 × 7.0 × 3.5 µm^3^.

By capturing a larger volume of bone-implant interface, PFIB-SEM tomography makes it possible to gain insights into the mineralization front(s) in newly forming bone, especially when examining osteoblasts and osteocytes. High *Z*-contrast features resembling cell bodies could be identified in the thin layer of resin between bone and the implant (Fig. 5A). These could correspond to either osteoblasts, indicating that in this area bone has grown towards the implant, or bone lining cells, suggesting the presence of an inactive surface. On the other hand, the cell becoming entrapped within the bone matrix (Fig. 4D–F, Fig. 5B) suggests that there is also a mineralization front directed in the opposite direction, i.e., moving away from the implant (Fig. 5B). At the bone surface close to this cell lacuna, globular elements of dense bone mineral could be observed (inset in Fig. 5B, Video S1). These features, often termed “mineral foci” or “calcospherulites”, have been reported at the mineralization front and in the early stages of mineralization^41–43^. Hence, their presence at the bone surface is a further indication of ongoing mineralization. The same bone surface also presents an area without any mineral foci, where the surface appears mostly smooth instead (Fig. 5B, arrowheads). As observed in previous work^15^, this location of the bone surface is likely quiescent and not an active formation/mineralization front^47^. As mineral foci grow during bone mineralization, they become the so-called “mineral ellipsoids”^11,17^ or “tesselles”^12^, which tessellate bone’s mesoscale structure in a crossfibrillar pattern ^12^. This is apparent also herein, as mineral ellipsoids can be noted in newly formed bone throughout the PFIB-SEM volume. The mineral ellipsoids present heterogeneous sizes and orientations (Fig. 5C–E, Video S1). Specifically, they are in register with each other only within small areas, but change in orientation abruptly from one region to another (Fig. 5E). Analogous changes in orientation have also been reported in the mouse tibia at the mineralization front of native bone (in the absence of an implant), where the orientation of the ellipsoids (therein called “tesselles”) reflected the angular offset of collagen bundles in adjacent bone (sub)lamellae^12^. This was also shown at the interface with an engineered porous implant in the rabbit tibia, leading to the hypothesis that this architecture contributes to bone toughness similarly to twisted plywood and Bouligand structures^15^.

Watershed segmentation of a sub-volume of the PFIB-SEM dataset identified 290 mineral ellipsoids, confirming their ellipsoidal shape in 3D (Fig. 5F,G). These ellipsoids presented a mean diameter of 739 ± 186 nm and 1.47 ± 0.35 µm in their transverse and longitudinal cross-sections, respectively (Fig. 5H), in agreement with previous findings^11,12,15,17^. This confirms the ubiquitous nature of mineral ellipsoids at biomaterial interfaces in yet another scenario, and specifically in the presence of altered bone metabolism and repair in the ovariectomized rat, which is a widespread preclinical model for postmenopausal osteoporosis.

Some areas with visibly larger ellipsoids, with transverse diameters up to 2.5 µm, were also identified from a qualitative inspection of the entire tomogram (Fig. 5D). Some brighter bands, without any ellipsoids distinguishable, were also noted (Fig. 5C, arrowheads). In these regions, ellipsoids may be so tightly packed that their individual peripheries are indistinguishable. These areas could also correspond to cement lines marking the position where different mineralization fronts meet. Size heterogeneity of mineral ellipsoids could be related to the mineralization stage and/or the rate at which bone formation proceeds. The dependence of mineral ellipsoid dimensions on mineralization progression has been shown in the tibia of wild type and *Hyp* mice, with ellipsoids enlarging as the distance from the mineralization front increases^12^. Conversely, some of the large mineral ellipsoids observed herein were relatively close to the mineralization front (Fig. 5D). However, in a group of ellipsoids segmented in a sub-volume of the tomogram closer to the mineralization front, no clear pattern in size variation with respect to the distance from the mineralization front was apparent (Fig. S2). The size of the ellipsoids in this sub-volume, with an average transverse diameter of 0.6 µm and longitudinal diameter of 1.1 µm, was overall comparable to that of the ellipsoids in Fig. 5F. Hence, it is possible that the large ellipsoids observed in some areas are a product of the altered bone repair due to ovariectomy and/or the local promotion of osteoblastogenesis by genistein. This may in turn cause an uneven deposition of extracellular matrix or irregular formation and spacing of mineral foci, resulting in larger ellipsoids in specific spots (although not necessarily in relation to the mineralization front) following mineral growth. On the other hand, it cannot be excluded that the contour between distinct ellipsoids is not well resolved in the dataset at times, making multiple neighbouring ellipsoids appear as one, large ellipsoid.

Since mineral ellipsoids have been largely unstudied to date, many aspects need to be investigated beyond their size and organization. For example, it remains undetermined how they interact with other structural features in bone’s meso-to-micro architecture, such as the LCN. We thus combined the segmentation of the ellipsoids with that of the LCN to gain an understanding of their spatial relationship. As osteoblasts become entrapped in their own mineralizing matrix (where mineral foci are growing into mineral ellipsoids), the LCN appears to spatially confine the growing ellipsoids by providing a boundary where mineral growth cannot proceed (Fig. S3). At the canalicular wall, mineralization instead is redirected around the periphery of the canaliculus rather than entering it. The growing ellipsoids then impinge on one another before a single ellipsoid can fully envelop a nearby canaliculus. Hence, canaliculi are observed to pass in between individual ellipsoids rather than penetrating the bulk of a single ellipsoid.

### Nanoscale: bone-implant interface and nano-osseointegration

**Figure 6:**
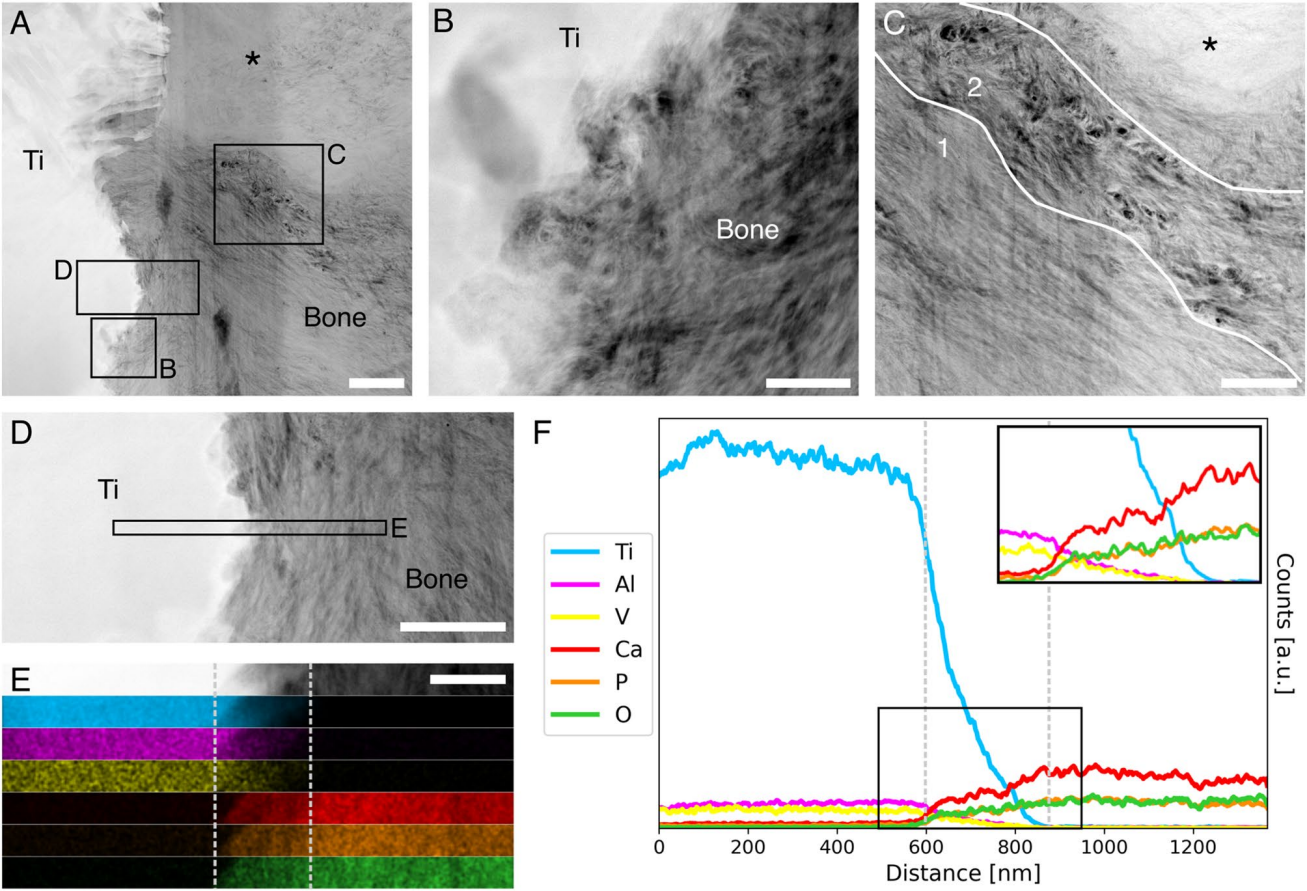
Bone-implant interface at the nanoscale. (**A**) HAADF-STEM overview image of the lamella of a bone-implant region of interest (ROI) prepared by FIB in situ lift-out. (**B**) Magnified image of an area of bone-implant contact occurring at the nanoscale, demonstrating nano-osseointegration. (**C**) Collagen fibrils change orientation abruptly from in-plane (region 1) to out-of-plane (region 2). The asterisk in the top region in A and C marks a brighter region, likely due to the higher thickness of the lamella in that area. (**D**) HAADF-STEM image of the bone-implant interface to provide a lower-magnification overview of the area where EDX acquisition was completed (marked by the rectangle). (**E**) From top to bottom, HAADF-STEM image of the area where EDX maps were acquired, and EDX maps of titanium (blue), aluminum (magenta), vanadium (yellow), calcium (red), phosphorous (orange), and oxygen (green). The interfacial area is marked by the grey dotted lines, and measures approximately 250 nm in width. (**F**) Elemental variation along a line directed from the implant towards bone (over the entire area of the maps in E), magnified in the inset. The grey dotted lines indicate the same interfacial zone marked on the maps in E. Scale bars are 1 µm in A, 200 nm in B and E, and 500 nm in C and D.

In the PFIB-SEM tomogram, a thin layer of resin and/or cracks were often present between bone and implant, hindering intimate contact between the two (Fig. 4B–F). These are mostly artifacts arising from implant retrieval and/or sample preparation. In particular, bone detachment from the implant can occur during retrieval^44,45^, while shrinkage during fixation and embedding often results in cracks at the bone-implant interface^46^. Nonetheless, some regions of close bone-implant contact, without any interposed resin or cracks, were identified in BSE-SEM images. An electron transparent lamella was prepared at one of said regions by FIB in situ lift-out (Fig. S4) and imaged by HAADF-STEM. This confirmed that bone-implant contact existed at the nanoscale (Fig. 6A,B). Collagen fibrils appeared to lack long-range registry, as groups of collagen fibrils co-aligned with each other and displaying the characteristic banding pattern of in-plane collagen were noted (Fig. 6C, region 1) alongside out-of-plane fibrils (Fig. 6C, region 2). This provided nanoscale confirmation of the abrupt changes in orientation observed in the PFIB-SEM tomogram in both this study and previous work^15^. Energy-dispersive X-ray spectroscopy (EDX) validated the gradual nature of the bone-implant interface at the nanoscale (Fig. 6D,E). Specifically, a decrease in content for implant elements (Ti, Al, V) was accompanied by an increase in Ca, P, and O (i.e., elements present in bone) (Fig. 6F). In the top region of the lamella, a brighter area, with not clearly distinguishable collagen fibrils, was observed (Fig. 6A,C). This area could mark a region with increased mineralization, similarly to the brighter bands in the PFIB-SEM tomogram (Fig. 5C, arrowheads). However, it is also possible that this represents an artifact due to a higher thickness in that portion of the lamella as a result of uneven thinning or an exaggerated wedge shape.

In this imaging and elemental analysis, the detection of genistein was not possible. The electrochemical layer-by-layer method produces an extremely thin coating of genistein (~ 40 nm), and its chemical composition, mostly consisting of hydrocarbons, is not sufficiently distinct from the organic compounds in bone to differentiate it in the EDX spectra and maps. Conversely, the roughened acid-etched surface is visible by HAADF-STEM and contributes to the nanoscale interlocking observed. While the characterization of the genistein coating and its quantitative effects on bone repair was beyond the methodological scope of this work, more tools can be implemented in our workflow to address these questions in the future. For example, imaging (SEM and atomic force microscopy) and spectroscopy techniques (e.g., Raman and Fourier transform infrared spectroscopy) can be used to determine structural and compositional properties of the coating, followed by larger in vivo studies to determine the role of local drug release on bone repair via immunohistochemistry to quantify the expression of relevant proteins^47^.

Despite the characterization approach adopted herein was inconclusive in determining the contribution of local genistein delivery on osseointegration, information gained across multiple length scales revealed a promising bone response despite the compromised bone repair conditions in the ovariectomized preclinical model. In particular, BSE-SEM showed new bone within the internal pores of the implant, confirming bone formation and ingrowth facilitated by the porous design, while HAADF-STEM and EDX validated that osseointegration occurred at the nanoscale. PFIB-SEM tomography, which combines imaging of large volumes with high spatial resolution, allowed us to bridge micro- and nanoscale levels in the characterization of osseointegration. This confirmed the existence of a well-developed LCN, validating 2D SEM images after resin cast etching. Notably, PFIB-SEM tomography revealed mineral ellipsoids throughout a large volume of bone-implant interface, corroborating the ubiquitous nature of these features at biomaterial interfaces, despite the compromised bone repair due to ovariectomy. Hence, this work emphasizes the importance of mesoscale characterization of osseointegration, which is especially relevant to determine the eventual role of mineral ellipsoids in the mechanical competence of bone interfacing with biomaterials, as well as the effect of systemic bone diseases and/or therapeutic agents on bone’s mesoscale architecture.

## Materials and methods

### Implant manufacturing

Ti-6Al-4V implants (diameter = 2 mm, height = 4 mm) were designed with 45% porosity in the mid-section (height = 1 mm) with 500 µm pores, together with a central solid strut for structural support (diameter = 300 µm) (Fig. 1). Implants were manufactured by L-PBF with a Renishaw AM 400 laser-modulated system (Renishaw, UK) using a hatch fill strategy with 200 W power, 75 µm point distance, 50 µs exposure time, 30 µm layer thickness, 65 µm hatch distance and 67° hatch increment angle, employing Ti-6Al-4V grade 23 feedstock powders with a particle size of 15–45 µm (AP&C, QC, Canada). Post-manufacturing, implants were cleaned by ultrasonication (15 min in ethanol, 15 min in acetone, and 5 min in deionized water), acid etched in 50% H_2_SO_4_ in deionized water at approximately 95 °C for 2 h, and functionalized with genistein by an electrochemical layer-by-layer coating technique [patent application BR 10 2021 019,134 1, Instituto Nacional da Propriedade Industrial, Brazil], following a method analogous to that described in^47^.

### Implant placement, and sample retrieval and preparation

Bilateral ovariectomy was performed on 3-month-old female Wistar rats (*n* = 2) as a model for impaired bone repair analogous to that occurring in osteoporosis^30^. At 30 days after ovariectomy, implants were placed in the tibia. The implant and surrounding tissue were retrieved *en bloc* after 28 days, a suitable time point to capture the peak of bone formation^48^ and observe bone remodeling in rats^49^. The samples were then fixed in 10% neutral buffered formalin, dehydrated in ethanol, embedded in polymethyl methacrylate resin, sectioned in half along the longitudinal axis of the implant, and polished prior to subsequent characterization.

### SEM survey

Embedded samples were mounted on aluminum stubs using carbon tape and silver paint, and sputter-coated with gold (~ 20 nm thickness). BSE-SEM images were acquired in a SEM instrument (JEOL 6610LV, JEOL, MA, USA) operated at high vacuum, 15–20 kV accelerating voltage, and a working distance of 10 mm. BSE-SEM images were used to identify areas of direct bone-implant contact for the preparation of the sample for STEM analysis.

### PFIB-SEM tomography

*Acquisition and reconstruction.* PFIB-SEM tomography was performed in a dual-beam Xe^+^ PFIB instrument (Helios G4 UXe, Thermo Fisher Scientific, MA, USA) using a 45° pre-tilt holder. A layer of carbon was deposited over a 70 × 25 μm^2^ ROI by ion beam deposition at 12 kV and 20 nA for the first 1 μm-thick layer, and at 65 nA for an additional 12 μm-thick deposit. Trenches were milled on both sides of the ROI using “cleaning cross-section” (CCS) milling patterns (30 kV, 0.20 μA and 15 nA currents) and in front of the ROI (30 kV, 0.20 μA and 4 nA currents). Fiducials were milled on the side of the exposed ROI cross-section and on the top of the ROI for tracking during tomography acquisition and for image alignment post-acquisition. The ROI cross-section was prepared for tomography acquisition by final CCS milling at 30 kV and 4 nA. Tomography was acquired in Auto Slice-and-View software, milling 30 nm slices by CCS at 30 kV and 4 nA, preceded by 4° rocking mill to minimize curtaining artifacts. Each slice was imaged by BSE-SEM at 1.2 kV and 0.20 nA at a working distance of 3.4 mm. The image stack was aligned using translation by Optical Flow (smallest step = 0.1%, Gaussian pyramid level = 3) and Linear Drift Compensation (maximum compensation factor), and smoothed by Gaussian blur (3D kernel, size = 7, sigma = 1.5) in Dragonfly (ORS, QC, Canada).

*LCN visualization.* Osteocyte lacunae and canaliculi were segmented based on pixel intensity in Dragonfly (ORS, QC, Canada). First, “bone + implant” and “resin + LCN” ROIs were segmented by Otsu thresholding, assigning the “bone + implant” ROI to the upper Otsu range (140.45–255). This ROI was further refined by selecting the 218–255 range to isolate the titanium implant (“implant” ROI). As this operation also included regions corresponding to charging artifacts, these were removed from the “implant” ROI using the “Process islands” operation to keep the largest object in the segmented region (i.e., the implant). The “bone” ROI was obtained by subtracting the “implant” ROI from the “bone + implant” ROI. A separate ROI, hereinafter referred to as “filled bone”, was created by adding all the unlabeled pixels within the “bone” ROI using the “Fill inner areas” operation applied in 2D in the x, y, and z direction, with a manual refinement using 2D/3D brushes in the “ROI painter” tool. The LCN was obtained by subtracting the “bone” ROI from the “filled bone” ROI, followed by manual addition/removal of mislabelled pixels/voxels using the 2D/3D brushes in the “ROI painter” tool. The LCN was further refined using the “Process islands” operation.

*Mineral ellipsoids segmentation.* Mineral ellipsoids in three distinct sub-volumes (7.5 × 7 × 3.5 µm^3^; 5.42 × 4.2 × 2.45 µm^3^; and 4.5 × 4.5 × 4.5 µm^3^) of the reconstructed PFIB-SEM dataset were segmented by Watershed segmentation in Dragonfly (ORS, QC, Canada). After a coarse intensity-based segmentation of the ellipsoids using the “Define range” tool, the “seeds” for the Watershed algorithm were identified with five iterations of an “Open” morphological operation (kernel size = 9–13) followed by the creation of a “Multi-ROI (6-connected)”. The “landscape” for the Watershed algorithm was generated as an inverted greyscale image stack from the initial coarse segmentation of the ellipsoids. For the size analysis, the transverse and longitudinal mean diameters were evaluated as the minimum and maximum Feret diameter, respectively. Statistical analysis was completed in Python 3.8.10.

### HAADF-STEM and EDX

An electron-transparent sample (lamella) of the bone-implant interface (Fig. S4) was prepared in a dual-beam FIB instrument (Helios 5 UC DualBeam, Thermo Fisher Scientific, MA, USA) equipped with a 30 kV Ga^+^ ion beam, a gas-delivery system (MultiChem), and a micromanipulator (EasyLift). The lamella was prepared by in situ lift-out following existing protocols^50^. Briefly, a 12 × 2 μm^2^ ROI was coated with tungsten by electron and ion beam depositions. Rough trenches were milled at 30 kV with 65 nA and 9.3 nA currents. The sample was attached to the micromanipulator with a tungsten deposit, lifted out, and attached to a copper grid. The lamella was thinned to electron transparency (~ 150 nm thickness) by CCS milling at 30 kV and progressively lower currents (0.79 nA, 0.43 nA, 80 pA, and 40 pA). Lastly, final polishing was completed at 5 kV and 68 pA to limit Ga implantation and amorphization. HAADF-STEM images and EDX maps of the lamella were acquired at 200 kV in a S/TEM instrument (Talos 200X, Thermo Fisher Scientific, MA, USA) equipped with four in-column silicon drift detectors (Super-X detector). EDX maps were acquired on 1000 frames at a dwell time of 10 µs per pixel.

### Resin cast etching and SEM

After BSE-SEM imaging, PFIB-SEM tomography, and in situ lift-out, the samples were removed from the aluminum stubs and polished to remove the gold coating. Samples were immersed in 37% phosphoric acid for 10 s, thoroughly rinsed with deionized water, immersed in 5% sodium hypochlorite for 5 min, rinsed with deionized water again, and left to air-dry overnight. The samples were mounted on aluminum stubs using conductive carbon and aluminum tape, and sputter-coated with gold (~ 20 nm thickness). Secondary electron SEM images were acquired in an SEM instrument (JEOL 6610LV, JEOL, MA, USA) operated at high vacuum, 7 kV accelerating voltage, and a working distance of 10 mm.

### Ethical approval

The animal experiments were approved by the Ethics Committee on the Use of Animals at UNESP (approval no. 00733–2020) and by McMaster University (AUP 22-09-32), and were conducted in full compliance with institutional and national regulations, and ARRIVE guidelines.

### Supplementary Information

Supplementary Information 1.

Supplementary Video 1.

## Data Availability

Data are provided in the main manuscript or in Supplementary Information.
