# Supplementary material for: Mesoscale characterization of osseointegration around an additively manufactured genistein-coated implant
Source: Sci Rep. 2024 Jul 3;14:15339. doi: 10.1038/s41598-024-66058-1 (PMC11222380; doi:10.1038/s41598-024-66058-1)
Supplement: Supplementary file 1 — Supplementary Information 1. [file 41598_2024_66058_MOESM1_ESM.pdf]

SUPPLEMENTARY INFORMATION

Mesoscale Characterization of Osseointegration Around an Additively Manufactured Genistein-Coated Implant

Chiara Micheletti[1,2], Liza-Anastasia DiCecco[1§], Joseph Deering[1§], Wanqi Chen[1], Ana Cláudia Ervolino da Silva[3], Furqan A. Shah[2], Anders Palmquist[2], Roberta Okamoto[4,5], Kathryn Grandfield[1,6,7]

[1] Department of Materials Science and Engineering, McMaster University, Hamilton, ON, Canada

[2] Department of Biomaterials, Sahlgrenska Academy, University of Gothenburg, Gothenburg, Sweden

[3] Department of Diagnosis and Surgery, São Paulo State University, Araçatuba Dental School, Araçatuba, SP, Brazil

[4] Department of Basic Sciences, São Paulo State University, Araçatuba Dental School, Araçatuba, SP, Brazil

[5] Research Productivity Scholarship (Process: 309408/2020-2), Araçatuba, SP, Brazil

[6] School of Biomedical Engineering, McMaster University, Hamilton, ON, Canada

[7] Brockhouse Institute for Materials Research, McMaster University, Hamilton, ON, Canada

[§] Equal contribution.

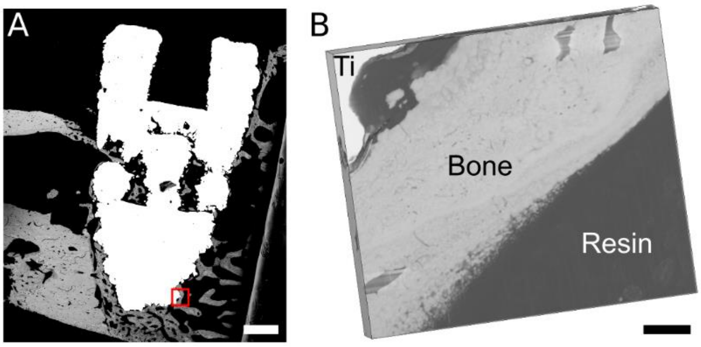

Figure S1. A) BSE-SEM overview image indicating the location of PFIB-SEM tomography acquisition (marked by the red rectangle). B) Visualization of the PFIB-SEM tomogram including resin and without false colouring, corresponding to Figure 4B-C. Scale bars are 500 µm in A and 10 µm in B.

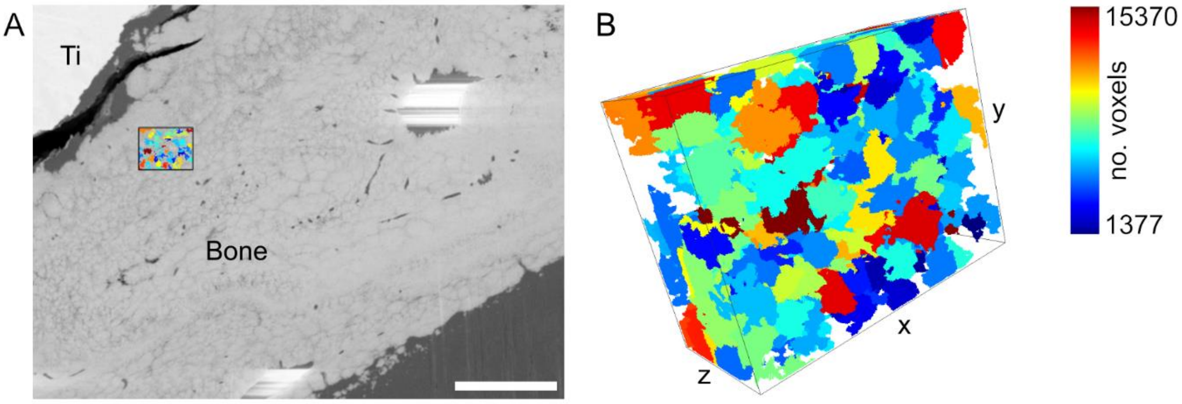

Figure S2. A) Region where mineral ellipsoids close to the mineralization front were segmented by the Watershed algorithm. B) 3D rendering of the segmented mineral ellipsoids, colour-coded based on size, expressed as voxel count (i.e., number of segmented voxel in each ellipsoid). The scale bar in A is 10 µm and the box in B is 5.4 × 4.2 × 2.5 μm^3.

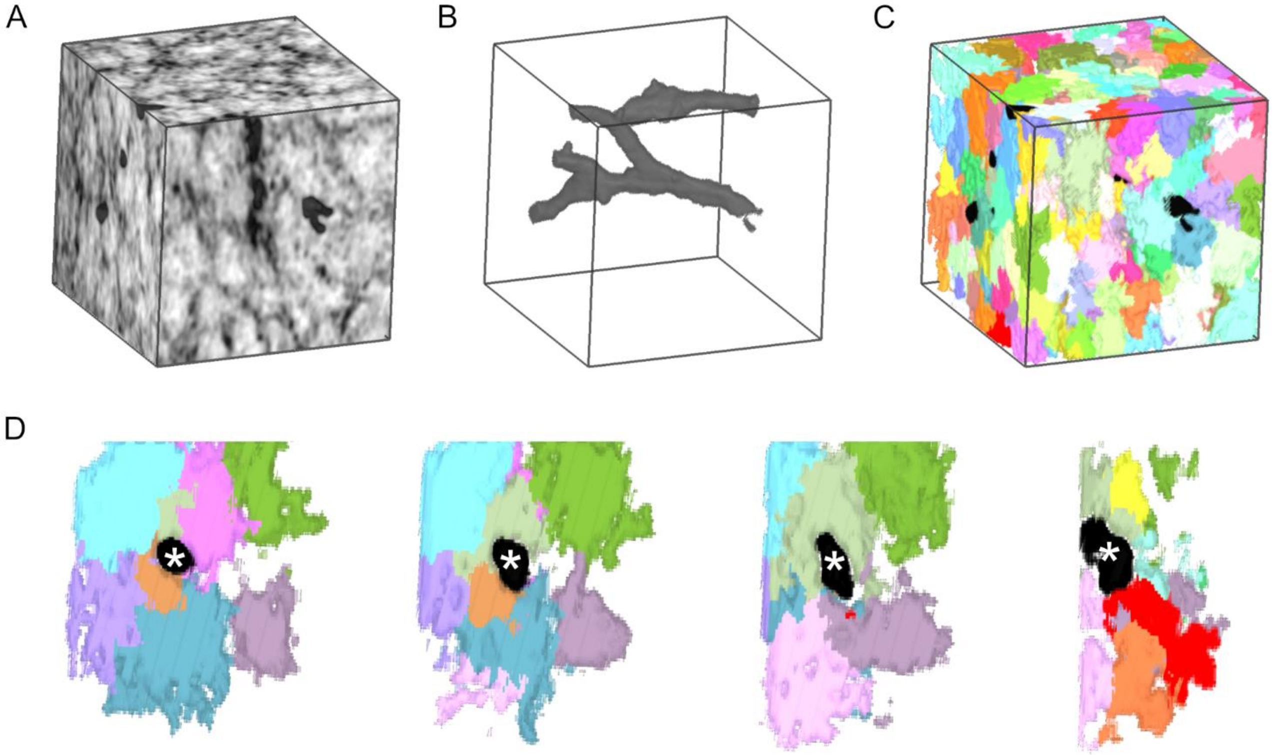

Figure S3. A) Sub-volume of the PFIB-SEM dataset where LCN and mineral ellipsoids were segmented. B) 3D rendering of the LCN segmentation (dark grey). C) Segmentation of the mineral ellipsoids with LCN shown in black. D) Successive cross-sections of a canaliculus (marked by *) permeating the space between mineral ellipsoids. The boxes in A-C are 4.5 × 4.5× 4.5 µm^3.

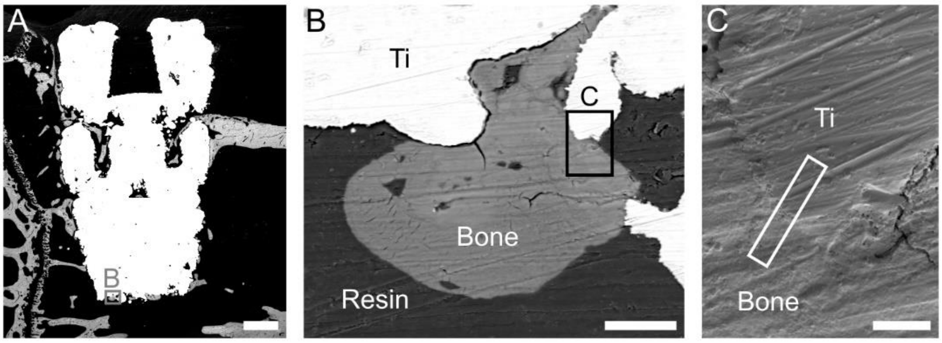

Figure S4. A) BSE-SEM overview image of the peri-implant space [Note: A is the same as Figure 3B, reproduced here to better show the site selection for STEM sample preparation]. B) BSE-SEM image of the area of bone-implant contact used for the preparation of the sample for STEM analysis. C) Secondary electron SEM image indicating the location selected for STEM sample preparation by FIB *in situ* lift-out (marked by the white rectangle). Scale bars are 500 µm in A, 30 µm in B, and 5 µm in C.

Video S1. Slice-through the PFIB-SEM tomogram showing slices in the image plane (xy). Scale bar is 10 µm.
